# Intravenous ivabradine versus placebo in patients with low cardiac output syndrome treated by dobutamine after elective coronary artery bypass surgery: a phase 2 exploratory randomized controlled trial

**DOI:** 10.1186/s13054-018-2124-8

**Published:** 2018-08-17

**Authors:** Lee S. Nguyen, Pierre Squara, Julien Amour, Daniel Carbognani, Kamel Bouabdallah, Stéphane Thierry, Caroline Apert-Verneuil, Aurélie Moyne, Bernard Cholley

**Affiliations:** 1 Critical Care Medicine, CMC Ambroise Paré, Neuilly-sur-Seine, France; 2 Anesthesiology and Critical Care Medicine, Hôpital de la Pitié-Salpétrière, AP-HP, and Université Pierre et Marie Curie, Paris, France; 3 Anesthesiology and Critical Care Medicine, Institut Mutualiste Monsouris, Paris, France; 4 Anesthesiology and Critical Care Medicine, Centre Cardiologique du Nord, Saint-Denis, France; 5 Institut de Recherches Internationales Servier, Suresnes, France; 6 Anesthesiology and Critical Care Medicine Department, Hôpital Européen Georges Pompidou, AP-HP, and Université Paris Descartes-Sorbonne Paris Cité, Paris, France

**Keywords:** Cardiac surgery, Low cardiac output syndrome, Cardiogenic shock, Postoperative atrial fibrillation, Coronary artery bypass graft, Systolic heart failure, Dobutamine, Sinus tachycardia

## Abstract

**Background:**

Low cardiac output syndrome (LCOS) is a severe condition which can occur after cardiac surgery, especially among patients with pre-existing left ventricular dysfunction. Dobutamine, its first-line treatment, is associated with sinus tachycardia. This study aims to assess the ability of intravenous ivabradine to decrease sinus tachycardia associated with dobutamine infused for LCOS after coronary artery bypass graft (CABG) surgery.

**Methods:**

In a phase 2, multi-center, single-blind, randomized controlled trial, patients with left ventricular ejection fraction below 40% presenting sinus tachycardia of at least 100 beats per minute (bpm) following dobutamine infusion for LCOS after CABG surgery received either intravenous ivabradine or placebo (three ivabradine for one placebo). Treatment lasted until dobutamine weaning or up to 48 h. The primary endpoint was the proportion of patients achieving a heart rate (HR) in the 80- to 90-bpm range. Secondary endpoints were invasive and non-invasive hemodynamic parameters and arrhythmia events.

**Results:**

Nineteen patients were included. More patients reached the primary endpoint in the ivabradine than in the placebo group (13 (93%) versus 2 (40%); *P* = 0.04). Median times to reach target HR were 1.0 h in the ivabradine group and 5.7 h in the placebo group. Ivabradine decreased HR (112 to 86 bpm, *P* <0.001) while increasing cardiac index (*P* = 0.02), stroke volume (*P* <0.001), and systolic blood pressure (*P* = 0.03). In the placebo group, these parameters remained unchanged from baseline. In the ivabradine group, five patients (36%) developed atrial fibrillation (AF) and one (7%) was discontinued for sustained AF; two (14%) were discontinued for bradycardia.

**Conclusion:**

Intravenous ivabradine achieved effective and rapid correction of sinus tachycardia in patients who received dobutamine for LCOS after CABG surgery. Simultaneously, stroke volume and systolic blood pressure increased, suggesting a beneficial effect of this treatment on tissue perfusion.

**Trial registration:**

European Clinical Trials Database: EudraCT 2009–018175-14. Registered February 2, 2010.

**Electronic supplementary material:**

The online version of this article (10.1186/s13054-018-2124-8) contains supplementary material, which is available to authorized users.

## Background

The low cardiac output syndrome (LCOS) is an acute circulatory disorder that may occur in 2% to 10% of patients after cardiac surgery [1–4]. When pre-operative left ventricular ejection fraction (LVEF) is altered, the prevalence of LCOS increases up to 20% [2]. In patients with coronary artery disease, the LCOS further impairs their ability to satisfy the myocardial demand in oxygen after coronary artery bypass graft (CABG) and is associated with a very high mortality rate [5]. The therapeutic management of LCOS involves dobutamine, an adrenoceptor agonist, which, having stronger beta- than alpha-adrenergic effects and resulting in a decreased afterload, is considered the inotrope of choice [6]. However, dobutamine also has a strong chronotropic effect that may compromise the expected hemodynamic improvement for two main reasons. First, the tachycardia decreases left ventricular filling time and may reduce stroke volume (SV) despite the improvement in ejection force [7]. Thus, as the increase in heart rate (HR) is dose-dependent, dobutamine global effect on cardiac output (CO) is often limited by the inherent tachycardia [8, 9]. Second, tachycardia is responsible for an increase in myocardial oxygen consumption that may aggravate myocardial oxygen imbalance [10]. These phenomena may also explain, in part, why dobutamine has been found as an independent risk factor for mortality after cardiac surgery in several studies [11, 12].

Ivabradine is a therapeutic agent that inhibits the sinus node I_f_ channel, which regulates the diastolic depolarization slope, therefore inducing a decrease in HR without affecting the conduction times (atrioventricular and intraventricular) or ventricular repolarization [13–16]. As a consequence, it may lower the HR while not affecting the ventricular ejection force. In addition, the increased time for ventricular relaxation and filling may improve SV, a feature of particular importance in patients presenting with reduced ejection fraction [17, 18]. Several case reports and controlled studies have shown that oral ivabradine administered in patients with LCOS decreased HR, improved global hemodynamics, and facilitated the weaning from dobutamine [19–23]. Moreover, the effects of the intravenous (i.v.) form of ivabradine have been documented in clinically stable patients presenting with systolic heart failure. In this population, ivabradine effectively decreased HR and increased SV along with left ventricular stroke work [24]. This study was designed to assess the hemodynamic effects of i.v. ivabradine in patients with LCOS treated with dobutamine after elective CABG.

## Methods

### Study design

This study was a phase II, multi-center, single-blind (sponsor not blinded) randomized placebo-controlled exploratory trial investigating the ability of i.v. ivabradine to control tachycardia in cardiac surgical patients whose LCOS was treated with dobutamine following elective CABG. Two university hospitals and three university-affiliated centers participated in the study, which was approved by ethics committees at these institutions. The trial (EudraCT: 2009–018175-14) was conducted in accordance with the principles of the Declaration of Helsinki, Good Clinical Practice guidelines, and local and national regulations. Written informed consent was obtained from all patients before any study-related procedures were performed.

The authors collected and interpreted data, drafted the manuscript, and made the decision to submit the manuscript for publication. Servier laboratories (Suresnes, France) sponsored the study and provided statistical support.

### Study participants

Patients were eligible if they were between the ages of 18 and 80 years and had a planned elective isolated CABG surgery with cardiopulmonary bypass (CPB), normal sinus rhythm, and a pre-operative echocardiographic LVEF between 20% and 40%. Patients were excluded if they presented any contraindication to ivabradine or severe comorbidities. All patients were monitored with a pulmonary artery catheter (PAC CCO, Edwards Lifesciences, Irvine, CA, USA), allowing investigators to measure CO and venous oxygen saturation of hemoglobin (SvO_2_) continuously. Patients were included if they presented an LCOS requiring dobutamine during the post-operative period. LCOS was defined by a cardiac index (CI) of less than 2.2 L.min^− 1^.m^− 2^ despite fluid resuscitation (guided according to CI response) and normothermia (36.5 ± 0.5 °C). The patients were subsequently randomly assigned in the study only if they developed a tachycardia with an HR of more than 100 beats per minute (bpm) in sinus rhythm.

### Randomization and treatment assignment

Patients were randomly assigned in a 3:1 ratio by using a computer-generated list to either ivabradine or matched placebo. Treatments were given as a fast infusion of 10 mg over 10 min, followed by a continuous slow infusion of 10 mg over 24 h. The slow infusion could be renewed for up to 48 h if the interruption criteria were not met. Treatment was interrupted as soon as the physician initiated the decrease in dobutamine infusion rate or in case of an adverse event.

Patients and physicians were blinded to the study treatment. An independent sponsor staff was aware of allocation groups in order to analyze the data and monitor adverse events.

At any time, dobutamine could be up-titrated according to the patient’s need (i.e., persistent LCOS) and epinephrine or norepinephrine could be administered in addition to dobutamine if deemed required by the physician in charge.

### Endpoints and criteria of judgment

The primary endpoint was the number and percentage of patients in each group in whom HR was reduced within the 80- to 90-bpm range for at least 30 min. The secondary endpoints included the changes in tissue perfusion reflected in (1) hemodynamic variables obtained by continuous monitoring—HR, CO, CI, and SV; systolic, diastolic, and mean blood pressure (SBP, DBP, and MBP, respectively); left ventricular stroke work index (LVSWI); SvO_2_; right atrial pressure (RAP); pulmonary capillary wedge pressure (PCWP); and urine output—and (2) biological parameters (creatinine clearance and serum lactate).

LVSWI was calculated as $LVSWI=\frac{CI}{HR}\times MBP\times 0.0144$, where 0.0144 represents the conversion factor used to express LVSWI in g.m/m^2^.

Safety endpoints, especially arrhythmias, were also monitored by using two-lead Holter monitoring up to 96 h (from a few minutes before dobutamine until 24 h after initiation of dobutamine decrease) and myocardial damage by using troponin Ic plasma levels.

The criteria for which the study drug had to be discontinued were bradycardia with HR of not more than 75 bpm, onset of post-operative atrial fibrillation (POAF) (except for spontaneously reversible episodes), sustained ventricular tachycardia, and conduction disturbances (atrioventricular or ventricular).

Because the duration of treatment varied among patients, we choose to present the data obtained at five specific time points: (1) at the time of dobutamine introduction because of LCOS (LCOS), (2) at the time of study treatment initiation (H0), (3) two hours after study treatment initiation (H2), (4) three hours after study treatment initiation (H3), and (5) immediately prior to study drug discontinuation (last value under treatment).

### Statistical analysis

Continuous values are presented as median and interquartile (25%–75%) ranges. Categorical values were compared by using chi-squared tests, and quantitative values were compared by using paired Wilcoxon signed-rank tests based on the non-parametric approach of Hodges and Lehmann for related samples. A *P* value of less than 0.05 was considered statistically significant.

## Results

### Baseline characteristics

Among 47 patients scheduled for CABG with CPB, 26 developed LCOS and received dobutamine, but only 19 developed sinus tachycardia with an HR of at least 100 bpm and were included in the study: 14 patients were randomly assigned in the ivabradine group and five in the placebo group (study flow chart in Additional file 1: Figure S1). Demographic pre-operative characteristics collected at the pre-selection visit are presented in Table 1. The two groups were similar regarding systolic heart failure severity, coronary artery disease history, and EuroSCORE. Patients in the ivabradine group tended to be older than patients in the placebo group.

**Table 1:** Baseline characteristics of patients

	Ivabradine group (*n* = 14)	Placebo group (*n* = 5)
Age, years	61 [59; 67]	54 [53; 59]
Male gender, n (%)	11 (79)	5 (100)
BMI, kg/m^2^	27.5 [25.8; 28.9]	26.2 [25.9; 29.7]
HR, bpm	73.5 [65.0; 89.0]	75.0 [67.0; 77.0]
SBP, mm Hg	121 [112; 130]	115 [114; 125]
DBP, mm Hg	71 [66; 73]	70 [65; 75]
EuroSCORE	5.5 [4.5; 6.5]	4.0 [2.5; 7.5]
LVEF, n (%)	31.5 [25.0; 38.0]	35.0 [27.0; 39.0]
History of MI, n (%)	9 (64.0)	2 (40.0)
History of PTCA, n (%)	5 (36.0)	0 (0.0)
History of CABG, n (%)	0 (0)	0 (0)
CAD severity, n (%)		
1 vessel	0 (0)	0 (0.0)
2 vessels	1 (7.1)	0 (0.0)
3 or more vessels	13 (92.9)	5 (100)
Dyslipidemia, n (%)	11 (78.6)	2 (40.0)
Hypertension, n (%)	9 (64.3)	3 (60.0)
Diabetes, n (%)	7 (50.0)	2 (40.0)
Heart failure, n (%)	7 (50.0)	3 (60.0)
Beta-blocker use, n (%)	13 (92.9)	5 (100.0)
eGFR, mL/min	82 [67; 110]	82 [76; 119]

Values presented as number (percentage) of patients and median [interquartile range].

Abbreviations: *BMI* body mass index, *CABG* coronary artery bypass graft, *CAD* coronary artery disease, *DBP* diastolic blood pressure, *eGFR* estimated glomerular filtration rate (calculated with Cockcroft-Gault formula), *HR* heart rate, *LVEF* left ventricular ejection fraction, *MI* myocardial infarction, *PTCA* percutaneous transluminal coronary angioplasty, *SBP* systolic blood pressure

### Primary endpoint

**Fig. 1:**
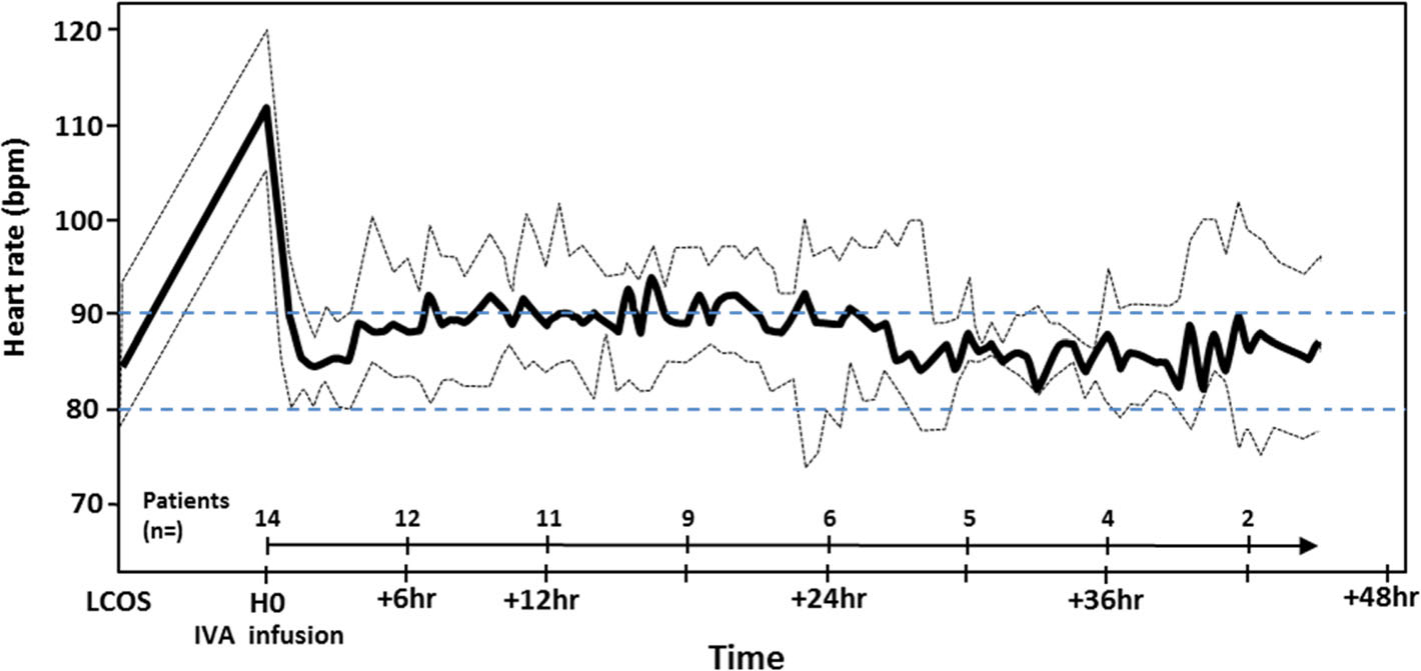
Heart rate variations in the ivabradine group. The bold line indicates median heart rate value, and the dotted lines delineate the first and third quartiles. Horizontal dashed lines indicate the target range for heart rate. Abbreviations: *bpm* beats per minute, *IVA* ivabradine (time of ivabradine initiation), *LCOS* low cardiac output syndrome (time of dobutamine initiation)

The primary endpoint was reached in 13 (93%) out of 14 patients of the ivabradine group and in 2 (40%) out of 5 of the placebo group. The introduction of ivabradine was followed by a quick and sustainable HR reduction from 112 [105–120] to 86 [78–96] bpm (*P* <0.001) (Fig. 1), compared with placebo group from 112 [104–120] to 104 [89–118] bpm (*P* = 0.125). Intergroup comparison was also significant (*P* <0.05). The median times to reach the HR target of less than 90 bpm were 1.0 h [0.5–1.5] for the ivabradine group and 5.7 h [5.7–5.9] for placebo (*P* = 0.13).

### Secondary endpoints

**Fig. 2:**
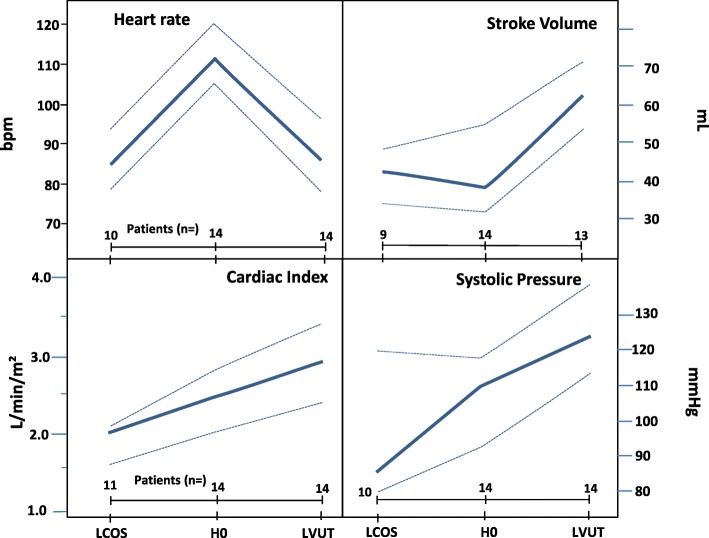
Hemodynamic variations in the ivabradine group (*n* = 14) between time of dobutamine initiation (LCOS), ivabradine initiation (H0), and cessation. Bold lines represent median values; dotted lines indicate quartiles 1 and 3. Abbreviations: *bpm* beats per minute, *LCOS* low cardiac output syndrome, *LVUT* last value under treatment

**Fig. 3:**
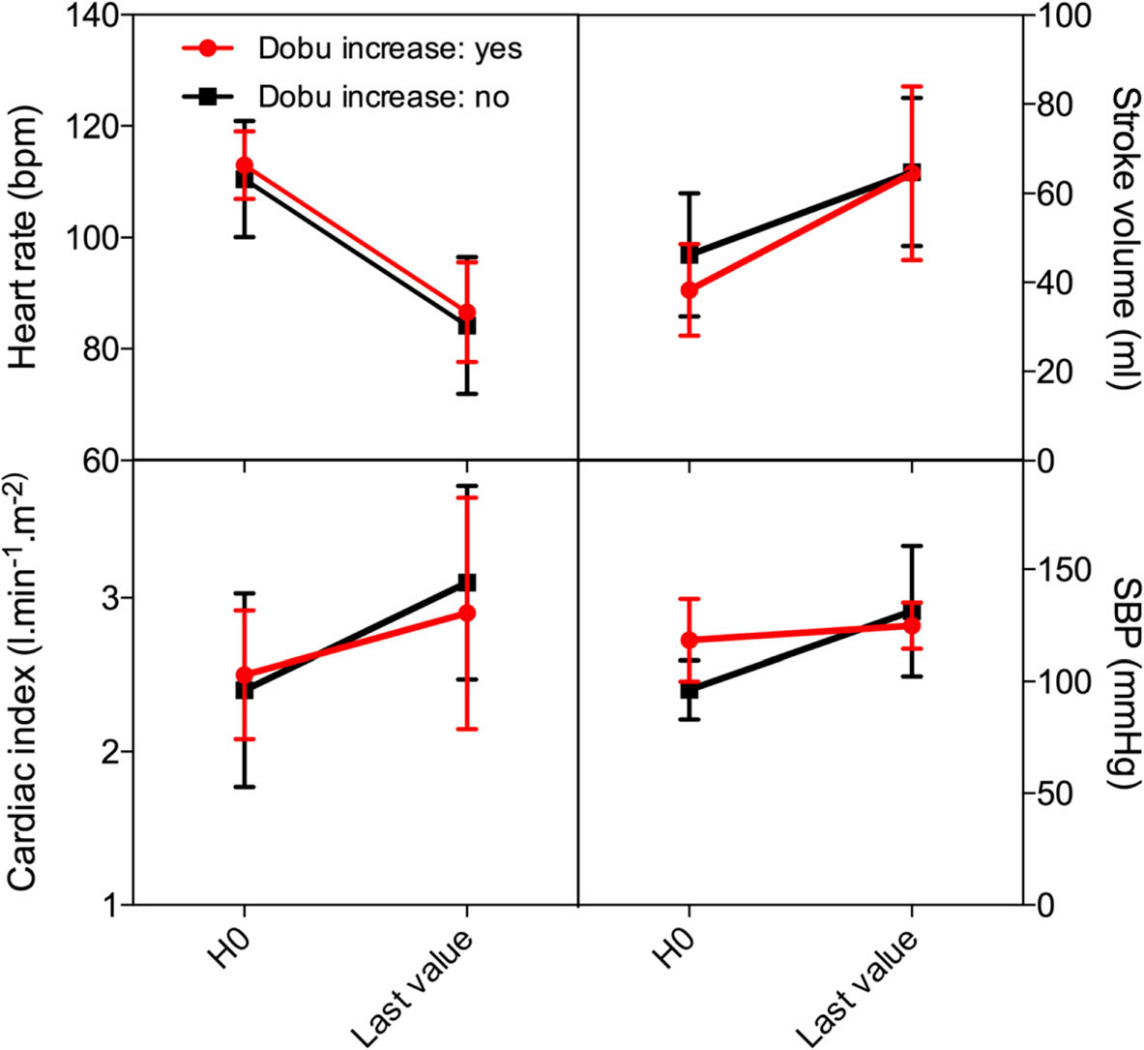
Comparison of hemodynamic effects of intravenous ivabradine between patients requiring an increase in dobutamine (*n* = 6) and those who did not (*n* = 8). Abbreviations: *bpm* beats per minute, *SBP* systolic blood pressure

Results are presented in Table 2. Changes between H0 and treatment cessation were observed in the ivabradine group for SBP (+19 [1–37] mm Hg, *P* <0.05); SV (+23 [11–37] mL, *P* <0.001); CO (+0.9 [0.2–1.7] l/min, *P* <0.05), CI (+0.6 [0.2–0.9] l/min/m^2^, *P* <0.05), and LVSWI (+9.1 [2.2–15.6] g.m/m^2^, *P* <0.05) (Figs. 2 and 3). Neither the changes within the placebo group nor the differences between ivabradine and placebo groups reached statistical significance.

**Table 2:** Hemodynamic variations between treatment initiation and cessation

		Ivabradine group (*n* = 14)			Placebo group (*n* = 5)		
		Median	[Q1–Q3]	*P* value^†^	Median	[Q1–Q3]	*P* value^†^
SBP, mm Hg	H0	110	[93–118]	<0.05	111	[92–128]	ns
	Last value	125	[114–139]		111	[90–121]	
DBP, mm Hg	H0	62	[55–71]	ns	51	[50–61]	ns
	Last value	59	[48–62]		55	[48–67]	
MBP, mm Hg	H0	78	[66–83]	ns	72	[68–84]	ns
	Last value	80	[75–82]		74	[60–83]	
SV, mL	H0	37	[32–55]	<0.001	55	[40–58]	ns
	Last value	60	[49–79]		62	[40–73]	
CO, L.min^−1^	H0	4.7	[3.6–5.4]	<0.05	5.4	[3.3–6.2]	ns
	Last value	5.3	[4.5–6.5]		5.9	[3.9–7.2]	
CI, L.min^− 1^.m^−2^	H0	2.5	[2.0–2.8]	<0.05	2.6	[1.7–3.1]	ns
	Last value	2.9	[2.4–3.4]		3.2	[2.4–3.4]	
LVSWI, g.m.m^− 2^	H0	20.0	[13.7–21.7]	<0.05	18.8	[13.9–31.9]	ns
	Last value	27.7	[18.5–32.4]		20.5	[17.4–34.7]	
SvO_2_ (%)	H0	71	[65–79]	ns	76	[75–85]	ns
	Last value	72	[57–79]		81	[76–84]	
PCWP, mm Hg	H0	18	[15–21]	ns	17	[16–17]	ns
	Last value	14	[12–18]		13	[11–18]	
RAP, mm Hg	H0	15	[10–17]	ns	14	[10–14]	ns
	Last value	11	[11–15]		14	[12–18]	
Diuresis, mL.kg^−1^.h^−1^	H0	1.20	[0.76–3.00]	ns	2.50	[1.42–2.69]	ns
	Last value	0.80	[0.39–1.08]		0.60	[0.29–0.82]	
eGFR, mL.min^−1^	H0	82	[67–110]	na	82	[76–119]	na
	Last value	76	[62–100]		62	[60–67]	
Lactate, mmol/L	H0	1.9	[1.3–2.8]	ns	2.1	[1.4–2.9]	ns
	Last value	1.7	[1.2–2.5]		1.2	[1.1–1.7]	
Troponin Ic, μg/L	H0	0.90	[0.36–3.00]	ns	2.00	[1.20–5.20]	ns
	Last value	2.70	[0.86–4.33]		1.00	[0.89–4.20]	

^†^Intragroup comparison between H0 and last value under treatment. Abbreviations: *CI* cardiac index, *CO* cardiac output, *DBP* diastolic blood pressure, *eGFR* estimated glomerular filtration rate (calculated with Cockcroft-Gault formula), *H0* before study treatment initiation, *Last value* last value under treatment, *LVSWI* left ventricular stroke work index, *MBP* mean blood pressure, *na* not appropriate due to the small sample size (seven patients in the ivabradine group and two patients in the placebo group), *ns* not significant, *PCWP* pulmonary capillary wedged pressure, *RAP* right atrial pressure, *SBP* systolic blood pressure, *SV* stroke volume, *SvO*_*2*_ venous blood oxygen saturation

The hemodynamic effects of ivabradine were quickly observed: in the ivabradine group, changes between H0 and H2 were similar to changes between H0 and last value under treatment: SBP (+9 [0–17] mm Hg), SV (+19 [9–35] mL), CO (+0.6 [− 0.1–1.7] l/min), CI (+0.4 [0–0.8] l/min/m^2^), and LVSWI (+10.6 [3.7–17.6]). Values for these variables at H3 were comparable to H2 (Additional file 2: Figure S2).

### Effect on concomitant catecholamine administration

Dobutamine infusion rate was increased in six patients (43%) with persisting LCOS in the ivabradine group as opposed to none in the placebo group. The resulting hemodynamic variations were similar in the subgroup of patients in whom dobutamine regimen remained unchanged (*n* = 8) (Fig. 3).

Cumulative dose of dobutamine was comparable between the two groups (955 [534–1088] mg in the ivabradine group versus 440 [236–905] mg in the placebo group, *P* = 0.18). This difference was due mostly to a difference in treatment duration: 23.7 [15.6; 38.0] hours in the ivabradine group versus 13.8 [3.3; 15.7] hours in the placebo group (*P* = 0.18). In the ivabradine and placebo groups, eight (57%) and two (40%) patients were treated with epinephrine, and four (29%) and one (20%) were treated with norepinephrine, respectively.

### Adverse events

Five patients (36%) developed POAF in the ivabradine group as opposed to none in the placebo group. One episode of bradyarrythmia occurred during the administration of ivabradine, requiring its cessation. The other four episodes occurred after the cessation of ivabradine infusion. In addition, ivabradine was associated with three cases (21%) of HR decrease below the threshold of 75 bpm, of which two (14%) required study drug cessation. Finally, one patient presented with several episodes of sustained ventricular tachycardia prior to and after ivabradine administration.

Troponin levels remained stable in both groups (Table 2). One patient in the ivabradine group developed post-operative septic shock and intestinal ischemia five days after study drug discontinuation. The patient died at day 7 following surgery, but this death was not considered related to the study drug. A second patient of the ivabradine group developed lung infection and subsequent septicemia 24 h following study drug termination. The patient recovered from this infection and ultimately was discharged from the hospital.

## Discussion

In this phase II exploratory multi-center randomized placebo-controlled trial involving patients with LCOS treated with dobutamine after CABG surgery, we observed that i.v. ivabradine infusion was associated with (1) a quick, durable, and significant control of HR in the target range of 80 to 90 bpm, (2) a significant increase in SV and SBP, and (3) a higher incidence of POAF and bradycardia.

### Hemodynamic effects of intravenous ivabradine

Like its oral counterpart, i.v. ivabradine was found to effectively reduce HR but with a shorter delay of action after treatment onset. This reduction was durable and allowed for a strict control of the HR in a pre-specified target range. In patients with LCOS, dobutamine is commonly used to improve oxygen delivery but induces tachycardia [25, 26]. It has been argued that most of the improvement in CO after dobutamine was due to the increase in HR [27]. However, the benefit of improving oxygen delivery may be counterbalanced by the deleterious effect of tachycardia on myocardial oxygen consumption. Indeed, some authors have observed that patients who received dobutamine after cardiac surgery had increased mortality in comparison with propensity-matched patients without dobutamine [11, 12]. Therefore, there is a sound rationale for controlling HR in CABG patients with post-operative LCOS treated with dobutamine. In the present study, patients were closely monitored to assess the safety of the dobutamine/ivabradine combination. We observed that the decrease in HR was associated with a concomitant increase in SV and CO. This suggests that the prolonged diastolic time improved left ventricular diastolic filling. The resulting improvement in SV was sufficient to compensate the decrease in HR and even increased CO.

These results confirm preliminary animal studies in which dobutamine combined with ivabradine allowed for a simultaneous contractile enhancement and prolongation of diastole, allowing for optimal filling and enhanced CO, in spite of a decrease in HR [22].

Because of the greater SV, SBP increased in the ivabradine group. MBP and DBP remained unaltered, indicating that systemic and left ventricular perfusion pressures were preserved. Overall, LVSWI increased and atrial pressures (RAP and PCWP) tended to decrease, suggesting a beneficial effect on biventricular end-diastolic congestion. The association of increased systemic blood flow, preserved mean arterial pressure, and reduced venous congestion in the systemic and pulmonary veins attests to an improved hemodynamic status. This corroborates the observations that renal function was maintained and that serum lactate did not increase significantly. We can assume that oxygen delivery was adequate and end-organ function was preserved, indicating that LCOS management was effective. These findings suggest that ivabradine did not interfere with a compensatory tachycardia but rather corrected the unwanted dobutamine-induced acceleration in HR.

These results were consistent and confirmed when assessing hemodynamic parameters at H2 and H3 after treatment initiation when most ivabradine patients reached target HR, emphasizing the causal role of ivabradine rather than the effect of time (as observed in the placebo group).

Indeed, ivabradine represents one of a few i.v. HR-decreasing agents without negative effects on inotropism, allowing its use in post-operative LCOS. Other existing therapeutic agents include digoxin and magnesium. Intravenous digoxin has the major drawback of presenting dose-dependent toxicity, including potential lethal arrhythmias, hyperkaliemias, and vasoplegic shock. Intravenous magnesium, though less deleterious, has a less significant effect on sinus tachycardia. Beta-blockers are part of the routine treatment for most patients with heart failure (all patients but one in our cohort) but, owing to their adverse effect on contractility and their antagonism with dobutamine, can rarely be administered in the post-operative period. Thus, ivabradine appears as a very promising tool offering an original alternative to control HR, even in patients with severely impaired left ventricular function.

### Catecholamine administration in patients receiving ivabradine

Epinephrine and norepinephrine usage was similar in the two treatment arms. Dobutamine regimen was increased by attending physicians in six ivabradine patients who remained in LCOS. Although we cannot rule out that controlling tachycardia might have reduced CO in this subgroup, we believe that HR reduction facilitated dobutamine up-titration. The fact that changes in HR, MAP, and SV were similar in the subgroup of patients in whom dobutamine was not increased supports the role of i.v. ivabradine in hemodynamic improvement independently from other concomitant treatments.

### Safety issues

As previously described for oral ivabradine in the SHIFT (Systolic Heart Failure Treatment with the I(f) Inhibitor Ivabradine Trial) [28], the i.v. counterpart was also associated with episodes of arrhythmia and bradycardia. Incident arrhythmias were all supraventricular. Higher dobutamine cumulative dose may be involved in the higher incidence of atrial fibrillation in the ivabradine group, as suggested by the fact that most episodes occurred after ivabradine cessation. These arrhythmias were resolved within a few days of standard treatment. On the other hand, bradycardia, as defined by an HR below 75 bpm, was easily addressed by discontinuing ivabradine. This high threshold for defining “bradycardia” was chosen to avoid leaving patients with LCOS in a low perfusion state. This may have prompted early ivabradine discontinuation in some patients for safety reasons despite the absence of clinical signs of hypoperfusion.

### Limitations of the study

The small number of patients is the main limitation to the validity of our observations. The inclusion criteria of this study were very restrictive in order to include a homogeneous population of patients and to avoid confounding factors. As a consequence, the number of inclusions was small but nevertheless appropriate to reach the primary objective: demonstrating the reduction of HR in the ivabradine-treated group. Inferential statistics were performed while taking into account the small sample size, allowing us to observe trends in the secondary hemodynamic endpoints. Consequently, adjusted statistical analysis could not be performed to rule out the confounding effects of concomitant catecholamine administration on hemodynamic changes. However, ad-hoc analyses showed that associated catecholamine administration was similar in the two treatment arms. However, even if subgroup comparison among ivabradine patients (a) with dobutamine increase and (b) without dobutamine increase showed similar results in hemodynamic variations, it is not possible to prove that the effect of ivabradine was independent from dobutamine dosage modifications.

Regarding the primary endpoint, we choose to make comparisons between baseline and the time of study drug cessation rather than at a fixed time point. The timing of study drug interruption differed among patients, attesting to the variability in LCOS severity. Indeed, many factors can influence LCOS (i.e., pre-existing cardiac dysfunction, CPB and aortic clamp durations, and quality of myocardial protection during bypass), which cannot be controlled for. Thus, we deliberately choose to make the comparisons between baseline and the time when LCOS was considered over in each patient (dobutamine weaning). This pragmatic approach allowed us to minimize the administration of study drug, which was important from a safety point of view, and to compare patients when they had reached a similar hemodynamic situation. However, it can be noted that changes between H0 and H2 (common to all patients) were similar to changes between H0 and the time of study drug cessation. Finally, because investigators were not always available when eligible patients underwent surgery, those who were included in this cohort are not strictly consecutive patients.

## Conclusion

This exploratory study showed that in a specific population of patients, those who had dobutamine-induced tachycardia after elective CABG, the administration of i.v. ivabradine significantly and quickly reduced HR without impairing CO and arterial blood pressure. Larger studies would be necessary to better assess the overall hemodynamic effects of i.v. ivabradine alone or associated with dobutamine.

## Additional files

Additional file 1: **Figure S1.** Study flow chart. Abbreviations: *HR* heart rate, *LCOS* low cardiac output syndrome. (PPTX 53 kb)

Additional file 2: **Figure S2.** Hemodynamic variations in the ivabradine group (*n* = 14) between time of dobutamine initiation (LCOS), ivabradine initiation (H0), and 2 and 3 h after ivabradine initiation (H2 and H3). Bold lines represent median values, and dotted lines indicate quartiles 1 and 3. *P* values are from Wilcoxon signed-rank test. (DOCX 98 kb)

## Abbreviations

bpm: Beats per minute
CABG: Coronary artery bypass graft
CI: Cardiac index
CO: Cardiac output
CPB: Cardiopulmonary bypass
DBP: Diastolic blood pressure
HR: Heart rate
i.v.: Intravenous
LCOS: Low cardiac output syndrome
LVEF: Left ventricular ejection fraction
LVSWI: Left ventricular stroke work index
MBP: Mean blood pressure
PCWP: Pulmonary capillary wedge pressure
POAF: Post-operative atrial fibrillation
RAP: Right atrial pressure
SBP: Systolic blood pressure
SV: Stroke volume
SvO_2_: Venous oxygen saturation of hemoglobin
