# Supplementary material for: Intravenous ivabradine versus placebo in patients with low cardiac output syndrome treated by dobutamine after elective coronary artery bypass surgery: a phase 2 exploratory randomized controlled trial
Source: Crit Care. 2018 Aug 17;22:193. doi: 10.1186/s13054-018-2124-8 (PMC6097391; doi:10.1186/s13054-018-2124-8)
Supplement: Supplementary file 1 — Figure S1. Study flow chart. Abbreviations: HR heart rate, LCOS low cardiac output syndrome. (PPTX 53 kb) [file 13054_2018_2124_MOESM1_ESM.pptx]

## Slide 1
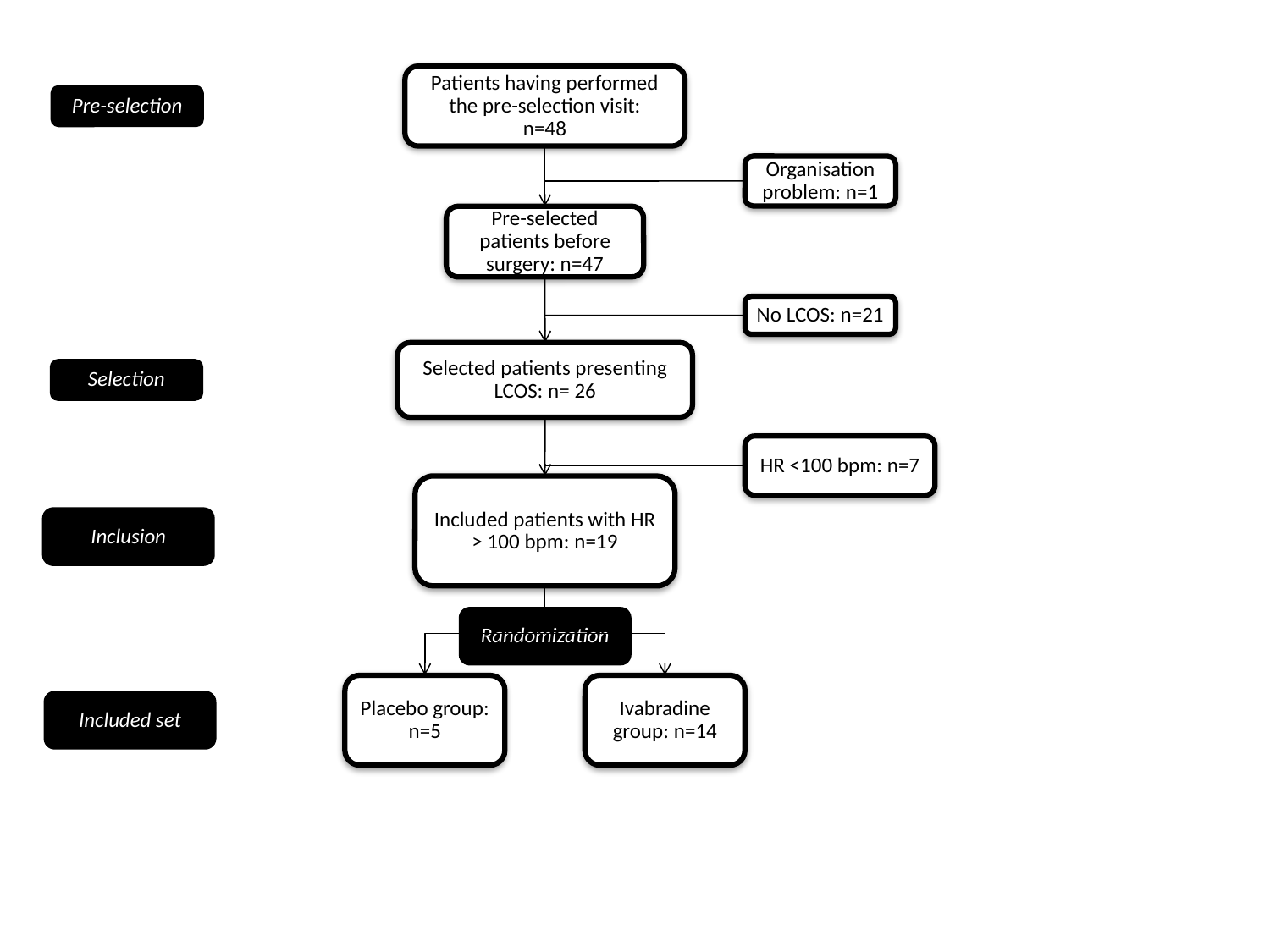

Patients having performed the pre-selection visit: n=48
Pre-selection
Organisation problem: n=1
Pre-selected patients before surgery: n=47
No LCOS: n=21
Selected patients presenting LCOS: n= 26
Selection
HR <100 bpm: n=7
Included patients with HR > 100 bpm: n=19
Inclusion
Randomization
Ivabradine group: n=14
Placebo group: n=5
Included set
