# Supplementary material for: Intravenous ivabradine versus placebo in patients with low cardiac output syndrome treated by dobutamine after elective coronary artery bypass surgery: a phase 2 exploratory randomized controlled trial
Source: Crit Care. 2018 Aug 17;22:193. doi: 10.1186/s13054-018-2124-8 (PMC6097391; doi:10.1186/s13054-018-2124-8)
Supplement: Supplementary file 2 — Figure S2. Hemodynamic variations in the ivabradine group (n = 14) between time of dobutamine initiation (LCOS), ivabradine initiation (H0), and 2 and 3 h after ivabradine initiation (H2 and H3). Bold lines represent median values, and dotted lines indicate quartiles 1 and 3. P values are from Wilcoxon signed-rank test. (DOCX 98 kb) [file 13054_2018_2124_MOESM2_ESM.docx]

**L/min/m²**

**bpm**

**mL**

**mmHg**

**Stroke Volume**

**Systolic Pressure**

**Heart rate**

**Cardiac Index**

**9**

**14**

**11**

**11**

**14**

**11**

**3.0**

**2.0**

**4.0**

**1.0**

**H0**

**LCOS**

**10**

**14**

**12**

**Patients (n=)**

**60**

**40**

**30**

**50**

**70**

**10**

**14**

**12**

**90**

**70**

**120**

**110**

**100**

**80**

**120**

**110**

**100**

**90**

**80**

**130**

**H2**

**H0**

**LCOS**

**H3**

**Patients (n=)**

**11**

**H3**

**12**

**11**

**12**

**H2**

**60**

*p* < 0.001

*p* = 0.088

*p* = 0.882

*p* = 0.005
